# Expired Platelet Concentrate Up-Cycling: Growth Factor-Rich Bioproduct Preparation for FBS Substitute

**DOI:** 10.3390/jcm12237345

**Published:** 2023-11-27

**Authors:** Eun Hye Lee, So Young Chun, Bo Hyun Yoon, Minji Jeon, Yun-Sok Ha, Jae-Wook Chung, Joonbeom Kwon, Jeongshik Kim, Dae Hwan Kim, Sang-Joon Park, Tae Gyun Kwon, Bum Soo Kim, Hyun Tae Kim

**Affiliations:** 1 Joint Institute of Regenerative Medicine, Kyungpook National University, Daegu 41566, Republic of Korea; eun90hye@gmail.com (E.H.L.); njiya120@naver.com (M.J.); 2 BioMedical Research Institute, Kyungpook National University Hospital, Daegu 41940, Republic of Korea; soyachun99@naver.com (S.Y.C.); bobo1904@naver.com (B.H.Y.); 3 Department of Urology, School of Medicine, Kyungpook National University, Daegu 41944, Republic of Korea; yunsokha@gmail.com (Y.-S.H.); jeus119@hanmail.net (J.-W.C.); tgkwon@knu.ac.kr (T.G.K.); 4 Department of Urology, Daegu Fatima Hospital, Daegu 41199, Republic of Korea; aziru98@gmail.com; 5 Department of Pathology, Ulsan Joongang Hospital, Ulsan 44667, Republic of Korea; kjspath@hanmail.net; 6 Department of Laboratory Animal Research Support Team, Yeungnam University, Daegu 42415, Republic of Korea; ikorando5@hanmail.net; 7 Department of Histology, College of Veterinary Medicine, Kyungpook National University, Daegu 41566, Republic of Korea; psj26@knu.ac.kr

**Keywords:** expired platelet concentrate (PC), growth factors, human stem cell culture, fetal bovine serum (FBS), medium supplement

## Abstract

Due to the short storage period, large quantities of platelet concentrate (PC) are expiring. The expired PC cannot be injected into a blood vessel, but the activity of bioactive molecules, especially growth factors, is still preserved. In this paper, we organized a process to obtain a growth factor-rich bioproduct for use as a supplement in human cell culture by optimizing freezing, thawing, and sterilization conditions. Each unit of PC displayed visual differences, diverse biochemical values, and growth factor concentrations. To minimize lot-to-lot variation, we pooled a minimum of 10 PC units. The concentrations of growth factors were maximized through five freeze–thaw cycles for 12 h at −80 °C for freezing and for 5 min at 36 °C for thawing. We used a cell strainer with 40 µm pores, followed by a 0.45 μm filter and a 0.22 μm filter sequentially to sterilize the bioproduct with minimizing loss. The obtained growth factors remained stable for 4–6 h at room temperature (23 °C), 24 h at 4 °C, and 12 months at −80 °C. Cellular responses to the growth factor-rich bioproduct were tested with primary human renal proximal tubule epithelial cells. The cells exhibited a significantly increased growth rate, compared to the fetal bovine serum (FBS)-treated control group. The cells maintained their characteristic cuboidal shape, and stem cells and renal progenitor cells also preserved their genetic characteristics during culture. Therefore, the growth factor-rich bioproduct isolated from expired PC through our process can be used as a medium supplement to replace FBS in human cell culture for clinical application.

## 1. Introduction

About 2.3% of blood products expire without being used, and platelet-related products account for the largest proportion of discarded blood (40.7%) [1]. The high expiration rate of platelet concentrate (PC) is attributed to its short five-day shelf life, compared to other blood products [2]. Expired PC cannot be injected into a patient’s blood stream, due to decreases in platelet number and function, as well as the risk of bacterial contamination [3]. However, PC’s constituents, including growth factors, cytokines, chemokines, anti-inflammatory factors, and antioxidant molecules, remain biological activation within platelet granules [4]. Thus, these bioactive molecules can serve as a source of human serum for in vitro cell culture and clinical cell therapy [5].

The platelet concentration in PC is 4–6 times (600–2400 × 10^9^) higher than that in whole blood [6]. Platelet alpha granules contain several growth factors, such as epidermal growth factor (EGF), platelet-derived growth factor (PDGF), transforming growth factor (TGF)-β1, insulin-like growth factor (IGF)-I, IGF-II, vascular endothelial growth factor (VEGF), endothelial cell growth factor (ECGF), and basic fibroblast growth factor (bFGF) [7]. These growth factors, both in terms of their concentrations and types, can induce clinical regenerative effects. Thereafter, PC can be used as a xeno-free growth supplement to replace fetal bovine serum (FBS), thereby minimizing immunological complications, preventing zoonotic infections, and avoiding ethical concerns related to the use of bovine fetuses [8].

Previous attempts to salvage expired PC included four steps: (1) thawing frozen PC, (2) separating supernatant and debris by centrifugation, (3) activating/lysing platelets through freeze–thaw cycles, and (4) sterilizing the supernatant using a filter before storage. However, the prescribed conditions, such as freeze–thaw temperatures, centrifugation time/speed, platelet activation/lysis method, the number of freeze–thaw cycles, and pore size for sterilization, vary across studies. Many of these studies also did not conduct microbiological analyses on the raw material, minimize lot-to-lot variation, provide a rationale for freeze–thaw conditions, optimize the sterilization process, or assess the stability of growth factors during storage.

Here, we present a new process for preparing a growth factor-rich bioproduct from expired PC, which involves the following steps: (1) analyzing the biochemistry of each unit, (2) minimizing lot-to-lot variation by pooling units, (3) optimizing freeze–thaw cycles and their temperatures, (4) optimizing a three-step sterilization process, and (5) assessing short-, medium-, and long-term storage stabilities. The obtained growth factor-rich bioproduct was subsequently used in human cell culture to assess its potential to replace FBS during in vitro culture.

## 2. Materials and Methods

### 2.1. PC Preparation

Expired PC was obtained from the biobank of Kyungpook National University Hospital (Daegu, Korea) after approval from the institutional review board (KNUH 2019-08-008-002). The expired PC was promptly transferred to the biobank and stored at −80 °C until needed. Table 1 lists the ID, blood type, unit number, and cryopreservation duration for the obtained samples. For the 27 frozen units, the thawing process involved immersing them in a water bath at 37 °C for 2 min with gentle shaking at 30 rpm. Following this, 1.5 mL of each melted product was centrifuged at 13,000 rpm for 30 min at 4 °C. The supernatant was then collected and filtered through a 0.22 μm low-protein binding filter (BD Falcon, Franklin Lakes, NJ, USA). The remaining melted product was mixed, distributed, and centrifuged at 3500× *g* rpm for 30 min at 4 °C. The supernatant was collected, and any remaining particulate matter was removed using a 40 μm cell strainer (BD Falcon). The aliquots were stored at −80 °C until they were needed. All samples were prepared in triplicate, and all experiments were repeated three times.

### 2.2. Assessment of Unit Variability in Visual Appearance, Biochemistry, and Growth Factor Concentration

One milliliter of each unit was placed in an e-tube to compare its turbidity and color, visually. The brightness was quantified with Image J (bundled with 64-bit Java 8, NIH, Bethesda, MA, USA). The average value was determined by measuring the brightness at five different spots within each tube. The control values were 255 for the white background and 0 for the black background.

Various biochemical parameters were measured in each unit using an automatic biochemical analyzer (Hitachi-720, Hitachi Medical, Tokyo, Japan). These parameters included total protein, albumin, bilirubin, creatine kinase, glucose, phosphate, aspartate transaminase (AST), alanine transaminase (ALT), lactate dehydrogenase (LDH), uric acid, blood urea nitrogen (BUN), creatinine, lipid profiles [total cholesterol (T-chol), triglyceride, high-density lipoprotein (HDL), and low-density lipoprotein (LDL)], immunoglobulin (Ig) A, IgG, and IgM.

Measurements of the following growth factors were carried out using enzyme-linked immunosorbent assay (ELISA) kits (R&D Systems, Minneapolis, MN, USA). The growth factors included epidermal growth factor (EGF), basic fibroblast growth factor (bFGF), insulin-like growth factor (IGF), vascular endothelial growth factor (VEGF), transforming growth factor-beta 1(TGF-β1), hepatocyte growth factor (HGF), platelet-derived growth factor (PDGF)-BB, and PDGF-AB.

### 2.3. Optimization of Freeze–Thaw Cycle Number, Temperatures, Incubation Times, and Storage Conditions

To optimize the freezing and thawing cycle numbers, PC (40 mL) was subjected to a freezing and thawing process. It was frozen at −80 °C for 12 h and thawed at 36 °C for 5 min. This cycle was repeated a total of 10 times. At each stage, 1 mL of supernatant was collected, and the concentrations of total protein and bFGF were measured. The precipitate formed during each cycle was separated by centrifugation at 3000 rpm for 30 min at 4 °C, and its weight was measured.

To optimize the freezing temperature, PC (40 mL) underwent freezing at either −80 °C or −20 °C for 12 h, followed by thawing at 36 °C for 5 min. This freezing and thawing cycle was repeated 6 times. At each stage of this process, 1 mL of supernatant was collected, and the concentration of bFGF was measured.

To optimize the thawing temperature, PC (40 mL) was frozen at −80 °C for 12 h and then thawed at different temperatures: 36 °C for 5 min, 23 °C for 10 min, 17 °C for 18 min, or 4 °C for 20 min. This thawing process was repeated 5 times, and the bFGF concentration in 1 mL of supernatant was measured at each stage.

### 2.4. Filtration Process

The prepared solution (40 mL) that underwent a freezing process at −80 °C for 12 h and thawing at 36 °C for 5 min, repeated 5 times (named as the bioproduct), was subjected to centrifugation at 3500 rpm at 4 °C for 30 min to separate the cryoprecipitate. The resulting supernatant was then filtered using a cell strainer with 40 μm pores, followed by additional filtrations using 0.4 and 0.22 μm filters.

### 2.5. Stability Assessment

Stability was assessed based on storage temperature and duration. An amount of 1 mL of bioproduct was subjected to short-term storage (10 min–24 h) at 23 °C, medium-term storage (1–13 days) at 4 °C, and long-term storage (1–12 months) at −80 °C. Subsequently, the concentrations of bFGF and VEGF were measured.

### 2.6. Cell Responses

Primary human renal proximal tubule epithelial cells (hRPTECs) were obtained from Lonza (CC-3190, Basel, Switzerland). They were cultured in renal epithelial cell basal medium (CC-3191) and REGM^TM^ SingleQuots^TM^ supplements (CC-4127). The supplements contained 5% FBS. The cells were grown on a tissue culture polystyrene plate (Costar, Northwest Washington, DC, USA). For the experiments, cells at passage four were utilized. The prepared growth factor-rich bioproduct, which had been stored for 1 month, was added to the culture medium at a final concentration of 5%, instead of FBS. Cells were then seeded at a concentration of 1 × 10^3^ cells per well in a 96-well plate with clear, flat-bottom wells (Corning, NY, USA). To each well, 100 μL of the medium was added. The recommended supplement-containing medium was employed as a control.

Cell morphology was examined on days 1, 3, 5, and 7 using an inverted microscope (Olympus, Tokyo, Japan). The cell proliferation rate was assessed at each time point through the use of an MTT assay kit (Roche, Basel, Switzerland).

The expression levels of various genes were quantified using real-time PCR. The genes examined included human kidney-specific progenitors (CD133 and CD24), a representative renal progenitor (Pax2), general physiological features (pan-cytokeratin), an endocytic clearance receptor (Megalin), ion channels (MUC-1), water channels (AQP1), a sodium-dependent glucose transporter (SGLT2), and dedifferentiation (E-cadherin, β-catenin, and vimentin) markers. The primer sequences used are shown in Table 2. The process involved RNA extraction and cDNA synthesis, which were performed using a cDNA synthesis kit (Invitrogen, Waltham, MA, USA). Real-time PCR was conducted with SYBR Green, following these conditions: 95 °C for 10 min, then 40 cycles of 95 °C for 10 s, 58 °C for 50 s, and 72 °C for 20 s. Gene expression levels were determined using the 2^−ΔΔCt^ method.

### 2.7. Statistical Analysis

Values were expressed as mean ± SD. The analysis of variance and post hoc tests were used to assess the biological activity data, where *p* < 0.05 indicated statistical significance. Differences between treatment and control groups were compared using the unpaired Student’s *t*-test.

## 3. Results and Discussion

### 3.1. Identification of Unit Variability in Visual Appearance, Biochemistry, and Growth Factor Concentration

**Figure 1:**
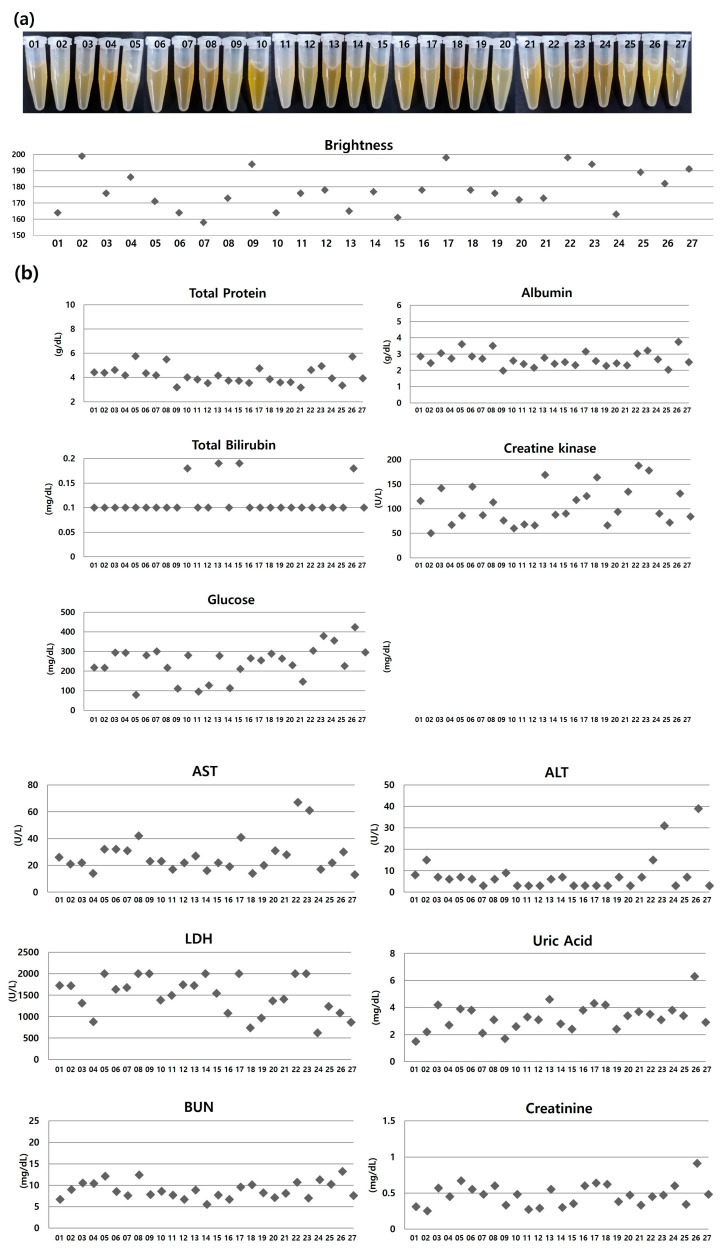

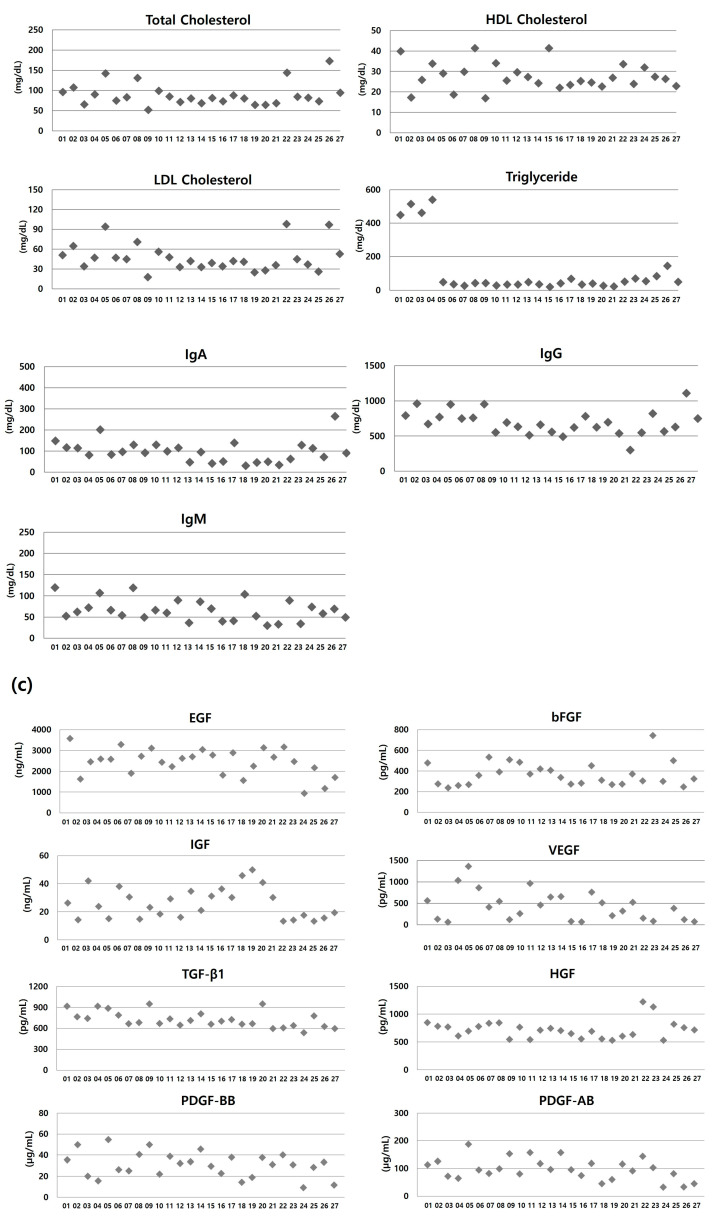
Variability in visual appearance, biochemistry, and growth factor concentrations across units. (**a**) Turbidity/color and brightness quantification, (**b**) biochemistry, (**c**) growth factors. Depending on the units, differences in visual appearance, biochemical values, and growth factor concentrations were observed. This experiment was repeated three times, and the mean value is expressed.

Each unit exhibited distinctive turbidity and color upon visual examination. The brightness values varied between 158 and 200, as depicted in Figure 1a, illustrating the visual diversity among each PC unit. Even though a single PC bag contained platelets from five blood donors of the same blood type and roughly 20% of the preservative solution [9], there were observable visual distinctions between each unit.

In the analysis of biochemistry, the values for total protein, albumin, total bilirubin, creatine kinase, glucose, phosphate, AST, ALT, LDH, uric acid, BUN, creatinine, total cholesterol, HDL cholesterol, LDL cholesterol, triglyceride, IgA, IgG, and IgM exhibited variabilities (Figure 1b). However, it is important to note that all of these values fell within the normal or acceptable range in all units [10]. This suggests that the expired PC would be acceptable biochemically.

Lot-to-lot variability was also observed for growth factor concentration, regardless of their type (Figure 1c). Notably, EGF and PDGF-AB exhibited relatively high coefficients of variability, with a ranges of 3000 ng/mL and 200 ug/mL, respectively, compared to other growth factors. This significant lot-to-lot variation could make it challenging to ensure consistent quality if PC bioproducts were to be commercialized. Thus, it is necessary to select an appropriate minimum unit number when preparing a pool as the starting material. For example, when measuring the average EGF value in five randomly selected units, the range was 2286.44–2718.65 pg/mL. However, by considering 10 units, the EGF levels ranged from 2401.87–2562.32 pg/mL, which is closer to the average range of 27 units (2429.34 ± 654 pg/mL). However, the variability will decrease with an increasing population, but it is not feasible to infinitely increase the number of units. Based on the above results, a minimum unit number of 10 was chosen to reduce lot-to-lot variability.

With this pool, the process was then optimized to manufacture a medium supplement that maximized the concentrations of growth factors.

### 3.2. Select the Optimial Freeze–Thaw Cycle Number

We employed the freeze–thaw method to activate platelets instead of thrombin, which would have led to increased production costs [11]. In this process, the freezing and thawing process may disrupt the membrane of the platelet granules, resulting in the release of adenosine diphosphate and calcium ions from delta granules into the plasma; thereby, the platelets are activated [12]. These platelets release numerous bioactive factors into the plasma. Repeated freeze–thaw cycles enhance the platelet activation and the release of growth factors more, but excessive cycles may diminish the activity of growth factors, due to their sensitivity to temperature [13]. Thus, it is necessary to determine the optimal number of repetitions that maximizes the release of growth factors, as well as preserving their activity. Also, the temperatures at which freezing and thawing could occur the activity of growth factors [14]. Hence, we optimized both the number of freeze–thaw cycles and the associated temperatures to achieve the desired results.

**Figure 2:**
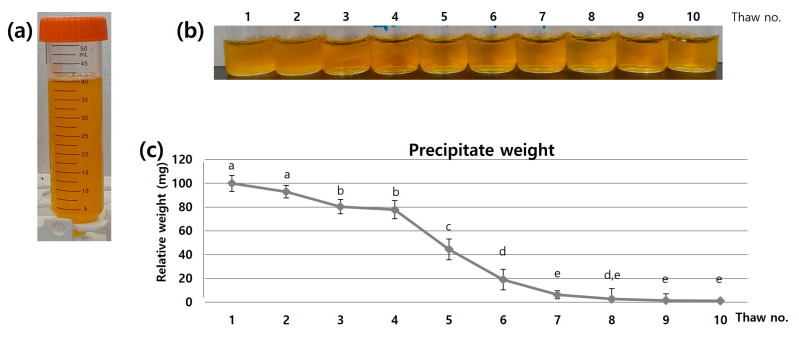

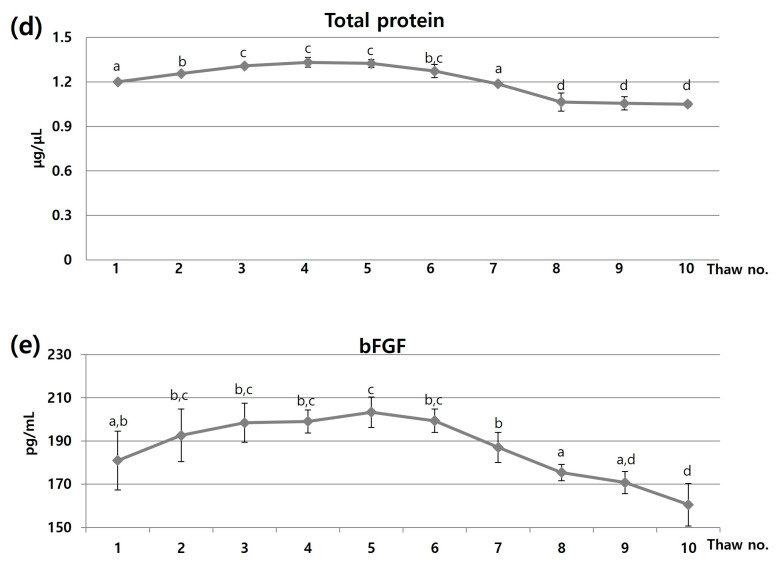
Optimization of freeze–thaw cycles. (**a**) Initial visual appearance, (**b**) intermediate appearances for 10 repetitions, (**c**) weight changes in the cryoprecipitate, (**d**) total protein, and (**e**) bFGF concentration changes in the PC supernatant. Five freeze–thaw cycles were selected to maximize the concentrations of growth factors. This experiment was repeated three times. Different letters in the chart indicate statistically significant differences (*p* < 0.05).

To ascertain the appropriate number of cycles, freezing and thawing were executed a total of 10 times. As the cycles progressed, the transparency of the supernatant increased (Figure 2a,b), due to the removal of the cryoprecipitate. The primary components of cryoprecipitate are debris from lysed platelets [14] and thermosensitive molecules that denature and precipitate during repeated freezing. Primary thermosensitive molecules include fibrinogen, factor VIII, factor XIII, von Willebrand factor, and fibronectin. These proteins have the propensity to precipitate from plasma at temperatures lower than 37 °C [15]. The cryoprecipitate persisted until around the sixth repetition (Figure 2c), which means that these repeated cycles are required to remove cryoprecipitates.

Based on the total protein analysis, the concentrations increased up to the fifth cycle, decreased until the eighth cycle, and remained constant thereafter (Figure 2d). The initial increase indicates that the concentration of plasma proteins (mainly albumin and globulin [11]) per unit volume increases following the removal of the cryoprecipitate. The decrease observed after the fifth cycle might be attributed to the denaturation of globulins caused by excessive temperature fluctuations [15]. The consistent concentration observed after the eighth cycle suggests that no further factors were affected by temperature variations beyond this point.

The analysis of growth factors indicated that the bFGF concentration increased up to the fifth cycle and subsequently decreased until the tenth cycle (Figure 2e). This observation indicates that the concentrations of growth factors in platelets are influenced by the number of freeze–thaw cycles. The process of freezing and thawing stimulates platelets to actively secrete growth factors. Furthermore, growth factors are passively released through platelet membrane destruction caused by freezing and thawing, as the formation of ice crystals in the platelet membrane during freezing triggers the bursting of platelets, resulting in the release of growth factors during thawing [12]. Thus, the concentrations of growth factors in PC increases due to the repeated freezing and thawing process. However, the stability of growth factors can also be affected by the activation of serum endotoxin and lipid peroxidase [13]. The activation of these peptide hydrolysis enzymes can lead to the degradation of growth factor peptides. Therefore, we opted for the fifth freeze–thaw cycle, as it maximized the concentrations of growth factors.

### 3.3. Select the Optimial Freeze and Thaw Temperatures and Incubation Times

**Figure 3:**
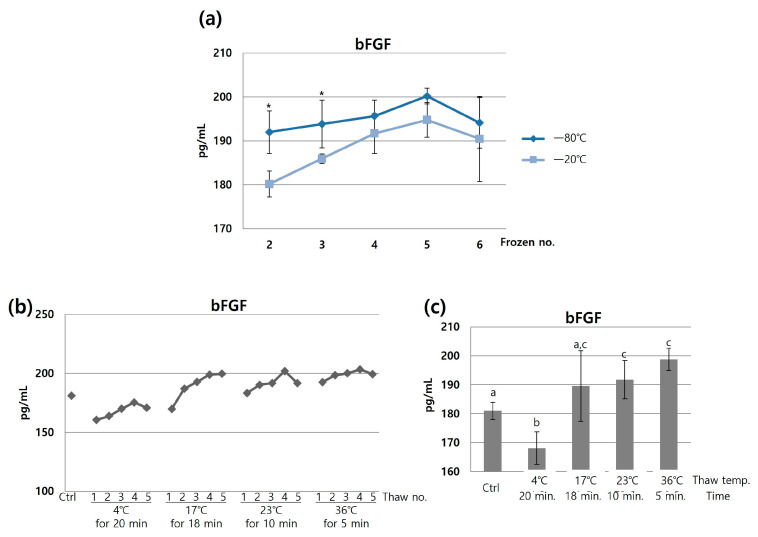
Optimization of freezing and thawing temperatures. (**a**) Comparison of freezing temperatures and (**b**,**c**) thawing temperatures. To maximize the growth factor’s concentration, the freezing and thawing temperatures were set at −80 °C and 36 °C, respectively. This experiment was repeated three times. The * or different letters in the chart indicate statistically significant differences (*p* < 0.05).

Next, we evaluated the stability of growth factors at different freezing temperatures. At both −80 °C and −20 °C, the concentrations of growth factors increased until the fifth freezing cycle and then decreased from the sixth cycle onwards. When comparing both groups, the concentrations were significantly higher in the samples frozen at −80 °C than those frozen at −20 °C (Figure 3a). This indicates that freezing at −80 °C was more effective in preserving the activity of growth factors, compared to freezing at −20 °C. Thus, the freezing temperature was decided to be −80 °C.

Following the optimization of the freezing temperature, the thawing temperature was tested at various levels, including 4 °C, 17 °C, 23 °C, and 36 °C. It was observed that the concentrations of growth factors increased as the number of thawing cycles increased (Figure 3b). Additionally, it was unexpectedly noted that the concentration of bFGF was particularly increased in the higher temperatures (Figure 3c). We initially expected that thawing at 4 °C would be the most effective way to preserve the activity of growth factors. However, due to the unexpected result of higher bFGF concentrations at higher temperatures, we began considering both the thawing temperature and the duration. At each temperature, the time required for thawing was 20, 18, 10, and 5 min, respectively. It is important to recognize that growth factors can degrade even at lower temperatures through enzymatic hydrolysis [12]. Consequently, it was determined that the thawing time can be a critical factor, as well as the temperature. Therefore, we revealed that rapid thawing at 36 °C proved to be more effective in preserving the activity of growth factors, compared to slow thawing at 4 °C.

### 3.4. Optimization of the Filtration Process

**Figure 4:**
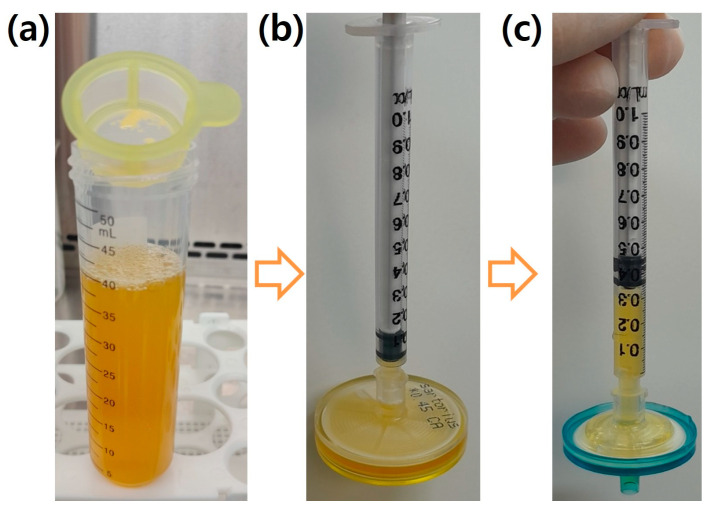
Optimization of the sterilization procedure. Sequential use of (**a**) a cell strainer (40 μm pores), (**b**) a 0.45 μm filter, and (**c**) a 0.22 μm filter. Through this procedure, the growth factor-rich bioproduct was sterilized with maximum harvest volume and minimum filter usage.

Sterilizing the bioproduct using a 0.22 μm filter before cell culture is a necessary step. However, this process often entails the use of several filters and results in a significant loss of the bioproduct. For instance, when filtering 1000 μL of the growth factor-rich bioproduct, 700 μL is used to wet the filter, 300 μL is infiltrated, and only 60 μL can be obtained through permeation. The filter is clogged and needs to be changed. Therefore, when filtering 40 mL of the growth factor-rich bioproduct, the final harvest volume will be 2.4 mL after using 40 filters. The low yield is primarily attributed to the presence of higher molecular weight components [11] in the bioproduct, which tend to block the pores in the 0.22 μm filter, thereby limiting the passage of the desired substances. Therefore, an efficient filtration process is essential to reduce the number of filters used and increase the final yield of the bioproduct. In this study, PC that underwent five freeze–thaw cycles was centrifuged at 3000 rpm (4 °C) for 30 min to separate the cryoprecipitate. The resulting supernatant, which contained the growth factor-rich bioproduct, was then filtered through a cell strainer with 40 μm pores to remove any floating lipids. This approach aimed to improve the efficiency of the filtration process and to enhance the yield of the bioproduct (Figure 4a). The supernatant was then filtered with a 0.45 μm filter (Figure 4b) and a 0.22 μm filter (Figure 4c), sequentially. In this process, the losses incurred due to the cell strainer, wetting the 0.45 μm filter, and wetting the 0.22 μm filter were 50 μL, 900 μL, and 700 μL, respectively. Consequently, from an initial 40 mL of bioproduct, approximately 38.5 mL of sterile extract could be recovered, with a minimal loss of only 1.5 mL and by using just three filters. This approach resulted in a remarkable 16-fold increase in harvest volume and a significant 13-fold reduction in filter usage, optimizing the filtration process.

### 3.5. Stability Evaluation According to Storage Conditions

**Figure 5:**
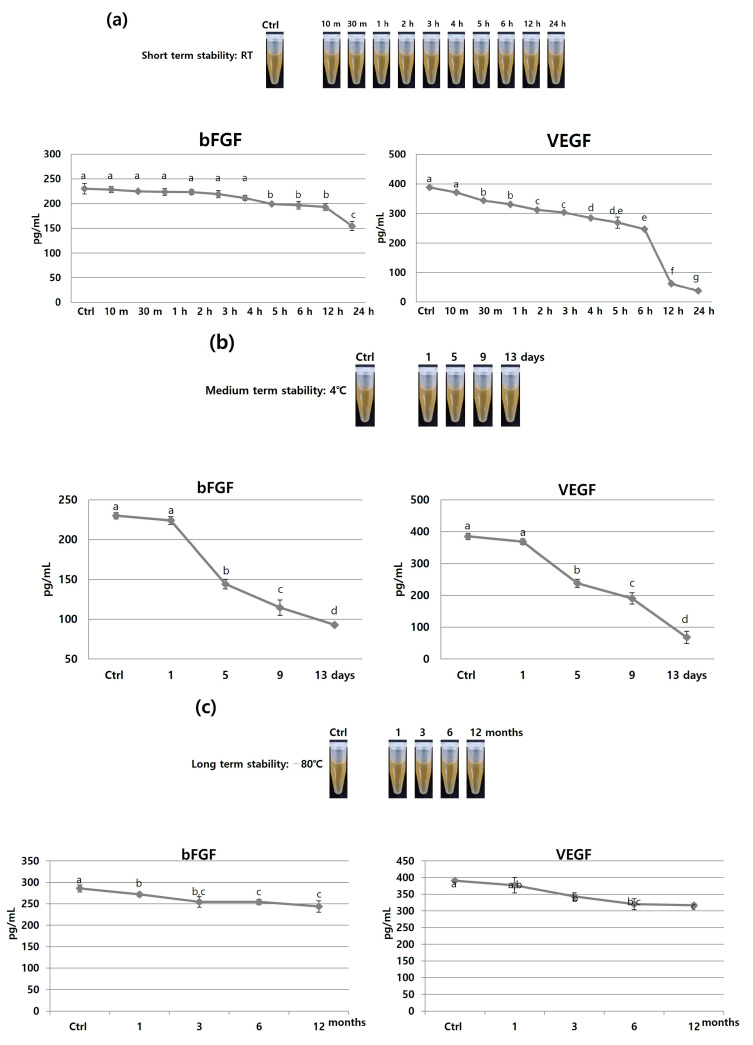
Growth factor stability analysis according to storage temperature and duration. bFGF and VEGF concentrations were measured following (**a**) short-term (24 h) storage at room temperature (23 °C), (**b**) medium-term (13 days) storage at 4 °C, and (**c**) long-term (12 months) storage at −80 °C. These growth factors were stable for 6 h at room temperature, 1 day at 4 °C, and 12 months at −80 °C. This experiment was repeated three times. Different letters in the chart indicate statistically significant differences (*p* < 0.05).

To assess the short-term stability (24 h), bioproducts were stayed at room temperature (23 °C). It was observed that the initial concentration of bFGF was sustained for 4 h, decreased to 15 pg/mL for the subsequent 12 h, and then significantly declined after 12 h. As for VEGF, its concentration gradually decreased by approximately 25 pg/mL at each time point, and it also significantly decreased after 6 h. This indicates that the stability of these growth factors diminishes over time at room temperature (Figure 5a). Taking into account that 80% of the initial concentration falls within an acceptable range, the growth factors can be considered stable at room temperature for a period of 4–6 h. However, it is important to note that this duration may vary depending on the specific growth factors being considered.

The medium-term stabilities (13 days) of growth factors at 4 °C were assessed. The concentration of bFGF decreased after the first day, but was not significantly reduced until the fifth day. Subsequently, it gradually declined, and by the 13th day, only approximately 40% of the initial bFGF concentration remained. Similarly, the VEGF concentration also decreased, with only about 17.67% of the original concentration remaining on the 13th day. This indicates that the stability of these growth factors decreased over the 13-day period when stored at 4 °C (Figure 5b). Hence, it is not advisable to store the bioproduct at 4 °C for more than one day, as the stability of the growth factors significantly diminishes over this time period.

The long-term stability (12 months) of growth factors at −80 °C was examined. While the concentrations of the growth factors gradually decreased over time, they remained within an effective range relative to the initial concentration. After 12 months of storage, the bFGF and VEGF concentrations were at 85% and 81% of their original levels, respectively. This indicates that activity of the bioproduct at −80 °C for up to 12 months is feasible, as the growth factors were stable within this period (Figure 5c). This makes the growth factor-rich bioproduct comparable to fresh frozen plasma, which can be stored and used for up to one year at −18 °C and for up to 36 months at −30 °C or lower [2]. However, it is essential to note that the storage duration was calculated based on one or two growth factors, the activity of which can be very different depending on the type of growth factor.

**Figure 6:**
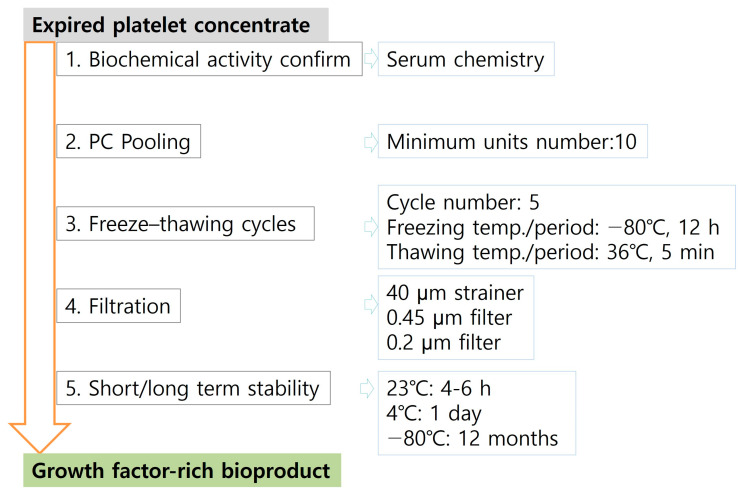
Summary of the growth factor-rich bioproduct isolate process from expired PC.

Thereafter, based on all of the all above results, we optimized the conditions of each process to prepare of the growth factor-rich bioproduct from expired PC (Figure 6).

### 3.6. Cell Responses

**Figure 7:**
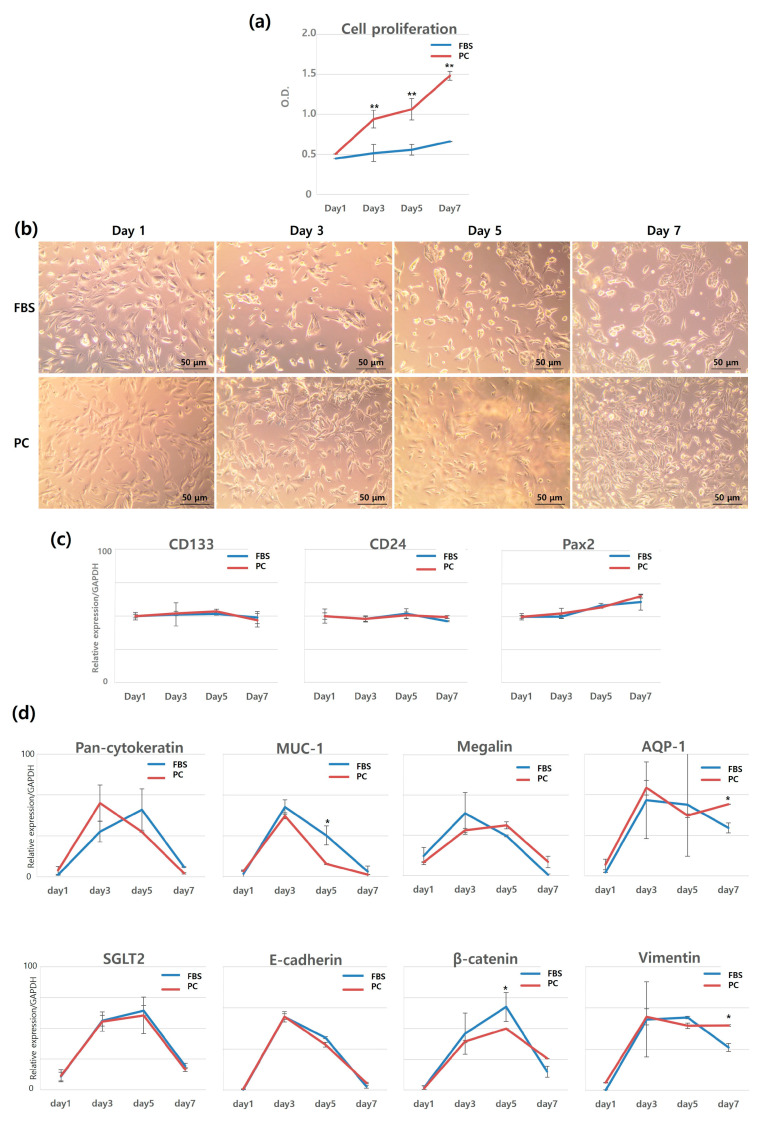
Effects of growth factor-rich bioproduct on human renal proximal tubule epithelial cells’ (hRPTECs) proliferation, morphology, and gene expression. (**a**) Cell proliferation analysis with MTT assay, (**b**) representative bright-field microscopic images, (**c**) renal progenitor genes, and (**d**) differentiation-associated genes expression of hRPTECs cultured in medium containing FBS or growth factor-rich bioproduct supplements. The growth factor-rich bioproduct promoted the proliferation of hRPTECs and maintained cell shape and stem cell characteristics. This experiment was repeated three times. (*, *p* < 0.05; **, *p* < 0.01). FBS, fetal bovine serum; PC, platelet concentrate that contains a large amount of growth factors by the established process.

The growth factor-rich bioproduct was subsequently utilized for the culture of hRPTECs. These cells are commonly used in studies related to kidney regeneration as a source of stem cells [14]. An analysis of cell growth rates showed that the medium containing the growth factor-rich bioproduct significantly stimulated the cell proliferation rate starting from the 3rd day of culture when compared to the medium containing FBS. By the 7th day, the cell proliferation in the bioproduct medium was approximately 2.24 times higher than that in the FBS medium (Figure 7a). Empirically, hRPTECs cultured with FBS do not show significant proliferation for 1 week. Usually, the rate gradually increases after 2 weeks. Early rapid growth due to the bioproduct can obtain the required number of cells (approximately 1 × 10^8^ or more) within a short period when applied as a cell therapy product, thereby reducing patient waiting time. 

Based on observations of cell morphology, the proliferated cells maintained their characteristic cuboidal shape (Figure 7b), which can serve as a marker for hRPTEC morphological characterization [16]. These results demonstrate that the bioproduct has the potential to significantly increase cell number while maintaining cell-specific morphology.

In the case of gene expression, the expressions of CD24, CD133, and Pax2, markers of human kidney-specific progenitor cells, showed similar patterns in both the experiment group and the control group (Figure 7c). Markers that track the differentiation process from progenitor cells to renal cells displayed transient significant differences in expression levels at various points during the culture period. However, when considering the overall duration of culture, there were no significant differences in the expression levels of these markers (Figure 7d). It is intriguing to note that, in contrast to the sustained expression of progenitor markers, most markers associated with differentiation and dedifferentiation (with the exception of AQP-1 and vimentin) tended to decrease on day 7 of culture in both groups. This suggests that both conditions, the growth factor-rich bioproduct and the FBS-containing medium, are conducive to maintaining the properties of renal progenitor cells rather than promoting their differentiation or dedifferentiation. This interpretation is further supported by the cell proliferation rate because fully differentiated cells typically cease proliferating, while progenitor or stem cells continue to proliferate. Therefore, the bioproduct derived from expired PC enabled hRPTECs to maintain their original cell morphology, exhibit rapid growth, and preserve the characteristics of stem cells/progenitor cells. These findings indicate that the prepared growth factor-rich bioproduct can serve as a substitute for xenogeneic serum, such as FBS, in human cell culture for clinical application.

## 4. Conclusions

From the expired PC, a growth factor-rich bioproduct was successfully obtained by pooling 10 units and subjecting them to five freeze–thaw cycles at −80 °C for 12 h and 36 °C for 5 min. This growth factor-rich bioproduct demonstrated a significant enhancement in the growth rate of cells while maintaining their cell morphologies and stem cell characteristics. This remarkable performance in cell culture makes it a suitable candidate to replace FBS in human cell culture and clinical applications.

## Figures and Tables

**Table 1:** Sample ID, blood type, unit number, and cryopreservation duration of the expired platelet concentrate.

Sample ID	Blood Type	Unit No.	Cryopreservation Duration (Days)
01 ~ 12	A	12	33
13 ~ 21	B	9	6
22 ~ 24	AB	3	19
25 ~ 27	O	3	30

**Table 2:** Primer Sequences.

Gene	Sequences	Type
CD24	5′-tca aca gcc agt ctc tc gt-3′ 5′-gac gtt tct tgg cct gag tc-3′	Kidney-specific progenitors
CD133	5′-ttc ttg acc gac tga gac cc-3′ 5′-tgg tct cct tga tcg ctg tt-3′	
Pax2	5′-tct ctc ctc tcc gct tct ct-3′ 5′-cga cag aga cgg aga ac-3′	Renal progenitor
Pan-Cytokeratin	5′-act tga caa ctt gca gca gg-3′ 5-caa tga tgc tgt cca ggt cg-3′	General physiological features
Megalin	5′-ctt gca act atc cga cct gc-3′ 5′-gga ccg ctt tca cat cca tc-3′	Epithelial cell properties
Muc-1	5′-tcc ttt ctc tgc cca gtc tg-3′ 5′-caa cca gaa cac aga cca gc-3′	
AQP1	5′-cca tca caa ctc tcc cca ct-3′ 5′-cca tca caa ctc tcc cca ct-3	
SGLT2	5′-ctc tct tcg cca gca aca tc-3′ 5′-cca ctc gaa tcc agc aac ag-3′	
E-cadherin	5′-aag ggg tct gtc atg gaa gg-3′ 5′-ggtgtt cac atc atc gtc cg-3′	Dedifferentiation
β-catenin	5′-gag ggt acg agc tgc tat gt-3′ 5′-aac gct gga cat tag tgg ga-3′	
Vimentin	5′-ctt tgc cgt tga agc tgc ta-3′ 5′-acg agc cat ttc ctc ctt ca-3′	
GAPDH	5′- tgt gtc cgt cgt gga tct ga-3′ 5′- cct gct tca cca cct tct tga-3′	House keeping

## Data Availability

Data are available upon request.

## References

[1] Kim B.C., Seo Y.I., Chai G.R., Shin J.W., Choi T.Y. (2011). Analysis of Discarded Blood Components at a University Hospital in Korea. Korean J. Blood Transfus..

[2] Gulliksson H., Sandgren P., Sjödin A., Hultenby K. (2012). Storage of platelets: Effects associated with high platelet content in platelet storage containers. Blood Transfus..

[3] Di Michele M., Van Geet C., Freson K. (2012). Recent advances in platelet proteomics. Expert. Rev. Proteom..

[4] Harmening D.M. (1997). Clinical hematology and fundamentals of hemostasis. Am. J. Clin. Pathol..

[5] Rauch C., Feifel E., Amann E.M., Spötl H.P., Schennach H., Pfaller W., Gstraunthaler G. (2011). Alternatives to the use of fetal bovine serum: Human platelet lysates as a serum substitute in cell culture media. ALTEX-Altern. Anim. Exp..

[6] Warnke P.H., Humpe A., Strunk D., Stephens S., Warnke F., Wiltfang J., Schallmoser K., Alamein M., Bourke R., Heiner P. (2013). A clinically-feasible protocol for using human platelet lysate and mesenchymal stem cells in regenerative therapies. J. Cranio-Maxillofac. Surg..

[7] Burnouf T., Goubran H.A. (2022). Regenerative effect of expired platelet concentrates in human therapy: An update. Transfus. Apher. Sci..

[8] https://www.abim.org/Media/bfijryql/laboratory-reference-ranges.pdf.

[9] Pallister C., Watson M. (2010). Haematology.

[10] Janes S. (2022). Encyclopedia of Respiratory Medicine.

[11] Khan N., Kurnik-Łucka M., Latacz G., Gil K., Saeed S.A. (2022). The Inhibitory Effect of Human Plasma Albumin and Haptoglobin on Platelet Aggregation and 5-HT Release. Folia Med. Cracov..

[12] Reid T.J., LaRussa V.F., Esteban G., Clear M., Davies L., Shea S., Gorogias M. (1999). Cooling and freezing damage platelet membrane integrity. Cryobiology.

[13] Bradley J.C., Simoni J.D., Bradley R.H., McCartney D.L., Brown S.M. (2009). Time-and temperature-dependent stability of growth factor peptides in human autologous serum eye drops. Cornea.

[14] Huang J., Kong Y., Xie C., Zhou L. (2021). Stem/progenitor cell in kidney: Characteristics, homing, coordination, and maintenance. Stem Cell Res. Ther..

[15] Simone S., Cosola C., Loverre A., Cariello M., Sallustio F., Rascio F., Gesualdo L., Schena F.P., Grandaliano G., Pertosa G. (2012). BMP-2 induces a profibrotic phenotype in adult renal progenitor cells through Nox4 activation. Am. J. Physiol. Ren. Physiol..

[16] Hoppensack A., Kazanecki C.C., Colter D., Gosiewska A., Schanz J., Walles H., Schenke-Layland K., Love H.D., Ao M., Jorgensen S. (2014). A human in vitro model that mimics the renal proximal tubule. Tissue Eng. Part. C Methods.

